# High‐frequency transcranial magnetic stimulation protects APP/PS1 mice against Alzheimer’s disease progress by reducing APOE and enhancing autophagy

**DOI:** 10.1002/brb3.1740

**Published:** 2020-06-26

**Authors:** Xia Chen, Guo‐Ying Dong, Lin‐Xiao Wang

**Affiliations:** ^1^ Department of Neurology Laboratory of Neurological Diseases Reproductive Medicine Centre Changzhou No. 2 People's Hospital The Affiliated Hospital of Nanjing Medical University Changzhou China

**Keywords:** Alzheimer's disease, ApoE, autophagic flux, cognitive impairments, repetitive transcranial magnetic stimulation

## Abstract

**Introduction:**

The repetitive transcranial magnetic stimulation (rTMS) has clinically wide application prospect of psychiatry and neuroscience, for its painless, noninvasive, and high efficiency. So far, rTMS has been used in the treatment of Alzheimer's disease (AD) but the underlying mechanism is not clear.

**Methods and Results:**

The APP/PS1 mice at 3‐month‐old were treated by 5 Hz high‐frequency (HF) rTMS for two weeks. After rTMS treatment, the AD‐like cognitive impairments of APP/PS1 mice were investigated subsequently, and molecular mechanisms underlying was further explored. The study showed that the 2‐week rTMS at 5Hz frequency improved cognitive impairments and AD‐like pathology (including a decrease in p‐Tau, APP, Aβ, and PP2A expression) of APP/PS1 mice. Although BDNF‐TrkB signaling was significantly enhanced, no differences of SYN, PSD95 and p‐AKT were observed in the brain of APP/PS1 mice. On the contrary, the LC3Ⅱ/LC3Ⅰ ratio was elevated with a significant reduction of ApoE and p62 in mice.

**Conclusions:**

rTMS exerts a potentially protective role in the prevention and treatment of AD by reducing ApoE expression and promoting autophagic flux, which provides a new insight into the mechanism of rTMS.

## Graphical abstract

This study aimed to investigate the functions and relevant mechanism of rTMS treatment on Alzheimer’s disease (AD) mice model. The study showed that the two‐week rTMS at 5Hz frequency improved cognitive impairments and AD‐like pathology of APP/PS1 mice via autophagy.

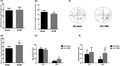

## 1 BACKGROUND

Alzheimer's disease (AD) is a progressive neurodegenerative disease that seriously endangers the health of middle‐aged and elderly people. With the aging of the population, the number of people suffering from AD is increasing rapidly, which is estimated to increase three times by 2050, and brings heavy burden to families and societies (Frankel et al., 2019; O'Shaughnessy et al., 2020). The characteristic pathological signs of AD are senile plaques formed by β‐amyloid (Aβ) deposition and neurofibrillary tangles formed by tau protein hyperphosphorylation, as well as neuron loss accompanied by glial cell proliferation. Cognitive disorders, personality and behavior disorders, insomnia, and autonomic nervous dysfunction are the mainly clinical symptoms of AD (Beauquis et al., 2014; Vergallo et al., 2018). For now, all drugs for AD just ameliorate the clinical symptoms, but have no prevention in the pathological process of AD. Thus, it is urgent to develop a new effective treatment to regulate the pathogenesis of AD.

As a noninvasive intervention, rTMS has developed into a promising choice for therapy and rehabilitation of neuropsychiatric diseases (Brunelin et al., 2014; Etoh et al., 2019; Hirakawa et al., 2018; Sabbagh et al., 2019). Both low‐frequency and high‐frequency rTMS have been proved to improve the cognitive function and synaptic plasticity of AD model in mice (Cotelli et al., 2011). Besides, the ability of language expression and understanding of moderate AD patients were significantly ameliorated after treatment with HF rTMS on the left dorsolateral prefrontal cortex, suggesting rTMS can alleviate cognitive impairments induced by AD indeed. However, the mechanism of rTMS in the treatment of AD is still unclear.

β‐amyloid (Aβ) begins to increase in the brain of APP/PS1 transgenic mouse (a classical AD mouse model) at about 3 months, and the plaque deposition can be detected around 6 months (Garcia‐Alloza et al., 2006; Zheng et al., 2017). The APP/PS1 mice at 3‐month‐old were treated by 5 Hz HF rTMS for 14 days in our study. The AD‐like cognitive impairments and AD‐related neuropathological features of APP/PS1 mice were investigated subsequently after rTMS treatment, and molecular mechanisms underlying was further explored. Our findings will provide new insights and research basis for the developing novel therapeutics for AD.

## 2 MATERIALS AND METHODS

### 2.1 Animals

APP/PS1 [B6C3‐Tg (APPswePSEN1dE9), MMRRC Cat# 034829‐JAX, RRID: MMRRC_034829‐JAX] double‐mutant transgenic mice (3‐month‐old) were purchased from Nanjing Biomedical Research Institute of Nanjing university. All mice were housed at room temperature of (22 ± 1°C) with plastic cages on a 12 hr/12 hr light/dark cycle. Food and water were available ad libitum. The animal experiments procedures were performed according to the Institutional Animal Care and Use Committee (IACUC) of Nanjing Medical University and approved by Changzhou No.2 People's Hospital Ethics Committee.

### 2.2 Application of HF rTMS

The APP/PS1 transgenic mice were randomly divided into two groups: sham‐rTMS APP/PS1 group (AD‐sham group, *n* = 15) and real rTMS APP/PS1group (AD‐rTMS group, *n* = 15). Mice in the AD‐rTMS group were treated with one session of 5 Hz HF rTMS daily for 14 consecutive days. Mice were awake and fixed in a specially made plastic cylinder according to our previous report (Zhang, Lu, Wang, Yun, & Zhou, 2019). The rTMS was delivered with a magnetic‐electric stimulator (CCY‐III, Wuhan Yiruide Medical Equipment Co., LTD.) with a round coil (6.5 cm diameter). The coil was held over the center of exposed head and parallel to the parietal bone of the mice. A total of 600 magnetic stimulation pulses consisting of 20 burst trains and 30 pulses each train at 5 Hz with 2‐s intertrain intervals were applied daily. The stimulation intensity represented 120% of the average resting motor threshold. The AD‐sham group was delivered by the cage of the coil positioning (perpendicular to the head scalp) with the same protocol of rTMS.

### 2.3 Novel objective recognition test

The novel object recognition (NOR) test was performed in a 50 × 50 × 50 cm white acrylic box. Each mouse was habituate in the box for two consecutive days without objects according to Shentu's study (Shentu et al., 2018). Next day the mice returned the arena from the same starting point, and two identical objects (old objects) were obtained for 5 min. The animal was later (after 2 or 24 hr, respectively) exposed to one of the old objects and a new object of a different shape and color. The video signal was transmitted to a computer in an adjacent room. After each trial, the objects and boxes were cleaned by 75% ethanol to eliminate odor cues. The recognition index was calculated as the time spent exploring the new object divided by the time exploring both objects.

### 2.4 Morris water maze test

Morris water maze (MWM) was used to detect assess learning and memory of mice (Morris, 1984). A round pool (120 cm × 50 cm) was filled by water (25 ± 1°C) with a white titanium dioxide. Then, an escape platform (10 cm × 15 cm) was placed in the center of one quadrant of the arena, 1–1.5 cm below the water surface. The walls of the test room were pasted with black‐and‐white extramaze cues. The trajectory of the mice was recorded by a video‐tracking camera mounted to the ceiling centrally above the pool. Once the platform was found, mice were allowed to sit on the platform for 10 s before being dried with a towel and returned to a heated drying cage. If the mice were not able to find the platform within 1 min, it was gently placed on the platform 60 s. Probe tests were performed after acquisition. The swimming path and times to reach target quadrant were recorded by a digital device connected to a computer.

### 2.5 Western blotting analysis

After behavior tests, total protein from the hippocampal tissues of the mice was collected for Western blotting. Hippocampal tissues were homogenized in radioimmuno‐precipitation assay (RIPA) and phenylmethylsulfonylfluoride (PMSF) lysis buffer (Beyotime) and incubation at 4°C for 30 min. The lysate was then centrifuged at 12,000 *g* for 10 min at 4°C to collect the supernatant. Protein concentration was determined according to BCA protein assay kit instructions (Beyotime). Equal protein sample was mixed with 5× loading buffer (Beyotime) and boiled for 10 min at 99°C.

A total of 50–80 μg protein samples were separated with 10% SDS‐PAGE and transferred onto a PVDF membrane (Millipore).The membranes were blocked with 5% nonfat milk for 1 hr at room temperature and then incubated with specific primary antibody diluted with TBST overnight at 4°C. The corresponding primary antibodies used were APP (Cell Signaling Technology Cat# 2452, RRID: AB_10694227), Phospho‐Tau (Ser199) Antibody (Cell Signaling Technology Cat# 29957, RRID: AB_2798984), Tau (phospho S396) antibody [EPR2731] (Abcam Cat# ab109390, RRID: AB_10860822), PP2A C Subunit Antibody (Cell Signaling Technology Cat# 2038, RRID: AB_2169495), Rabbit Anti‐PSD95 Polyclonal Antibody (Cell Signaling Technology Cat# 2507, RRID: AB_561221), Synaptophysin antibody [YE269] (Abcam Cat# ab32127, RRID: AB_2286949), BDNF antibody [EPR1292] (Abcam Cat# ab108319, RRID: AB_10862052), Phospho‐Akt (Ser473) Antibody (Cell Signaling Technology Cat# 9271, RRID: AB_329825), Akt Antibody (Cell Signaling Technology Cat# 9272, RRID: AB_329827), Rabbit Anti‐LC3B Polyclonal Antibody (Cell Signaling Technology Cat# 4108, RRID: AB_2137703), ApoE (pan) (D7I9N) Rabbit mAb antibody (Cell Signaling Technology Cat# 13366, RRID: AB_2798191), alpha‐Tubulin Antibody (Cell Signaling Technology Cat# 2144, RRID: AB_2210548), β‐Actin (8H10D10) Mouse mAb antibody (Cell Signaling Technology Cat# 3700, RRID: AB_2242334), Tau antibody [TAU‐5]—BSA and Azide free (Abcam Cat# ab80579, RRID: AB_1603723), SQSTM1/p62 Antibody (Cell Signaling Technology Cat# 5114, RRID: AB_10624872), TrkB antibody (Abcam Cat# ab18987, RRID: AB_444716), Recombinant Anti‐beta Amyloid 1‐42 antibody (Abcam Cat# ab201060, RRID: AB_2818982). The membranes were washed with TBST three times next day and incubated with secondary antibodies for 1 hr at room temperature. The protein was scanned with enhanced chemiluminescence kit (ECL, Thermo). Quantity‐one software (BIO‐RAD) was used to analysis the density of band.

### 2.6 Statistical analyses

Data were presented as mean ± standard deviation (*SD*). Statistical analyses were performed using SPSS 21.0 (SPSS, RRID: SCR_002865). Statistical significance was defined as *p* < .05.

### 2.7 Restoration of rTMS on learning, memory, and cognitive function of APP/PS1 mice

**FIGURE 1:**
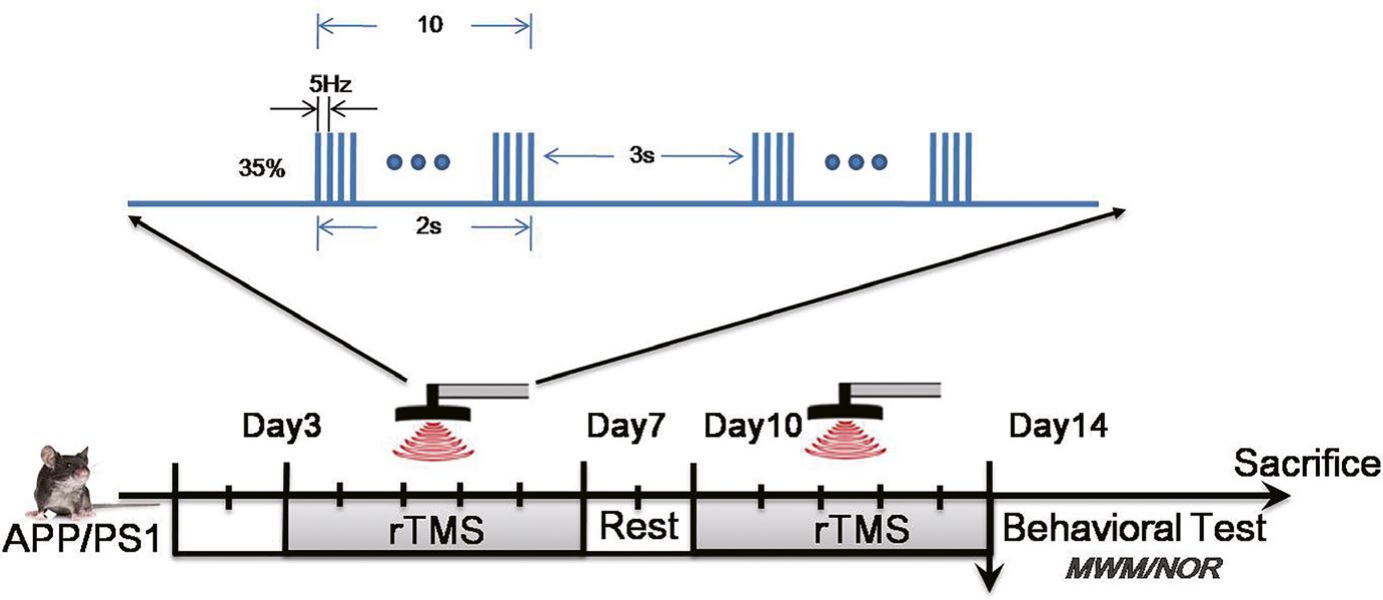
HF rTMS protocols delivered for 14 consecutive days. A total of 600 magnetic stimulation pulses consisting of 20 burst trains and 30 pulses each train at 5 Hz with 2‐s intertrain intervals were applied in each day

**FIGURE 2:**
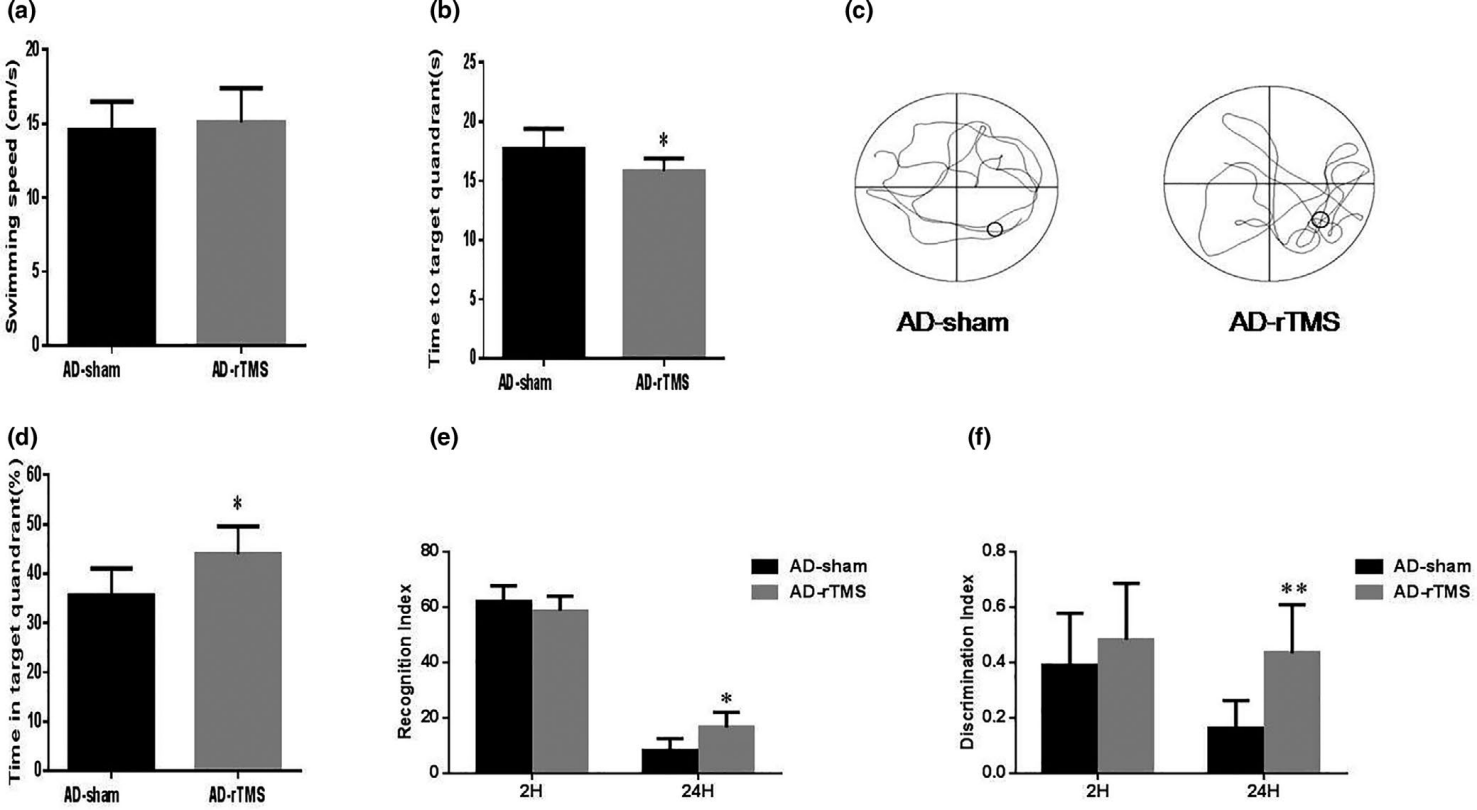
High‐Frequency rTMS ameliorates spatial memory and cognitive dysfunctions in APP/PS1 mice. Morris water maze (MWM) test was performed to assess learning and memory functions using this spatial reference memory task (a–d). (a) The mean swimming speed of each group. (b) The escape latency of MWM. (c) Illustrative images of mouse trajectories in the MWM recorded by computer. (d) The time in the target quadrant after removing the platform. The NOR (e, f) experiment was performed after rTMS treatment. **p < .01, *p < .05

After 14 days of consecutive intervention with HF rTMS (Figure 1), behavioral experiments (MWM and NOR) were used to evaluate the improvements of rTMS on learning, memory, and cognitive function of APP/PS1 mice. Compared with the AD‐sham group, rTMS treatment had no effect on the swimming speed of APP/PS1 mice (Figure 2a), indicating that rTMS has no influences of motor function. However, the escape latency of the rTMS group was markedly shortened, and the time spent in the target quadrant was significantly increased (Figure 2b–d), which implied that rTMS recovered the spatial learning and memory defects of APP/PS1 mice. In addition, a significant elevation was observed after rTMS treatment and the NOR index (Figure 2e,f) compared with the sham animals. The above results revealed that HF rTMS alleviated the cognitive impairment of learning and memory in AD mice.

### 2.8 Effect of rTMS on neuropathological features of AD in APP/PS1 mice

**FIGURE 3:**
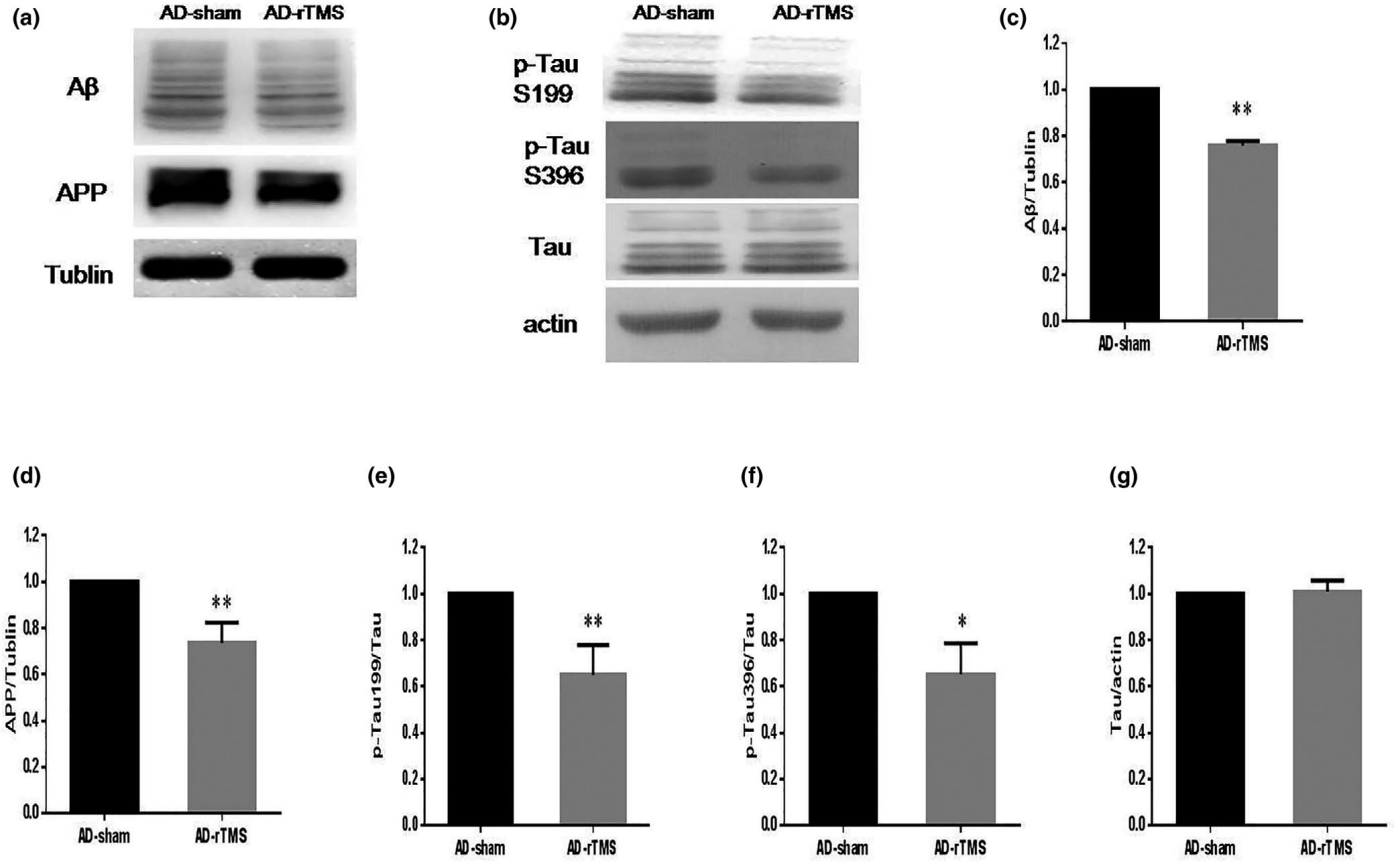
rTMS rescues AD‐related lesions in APP/PS1 mice. Hippocampal protein expression of Aβ, APP, and p‐Tau was detected by Western blots (a, b). The quantification graph was shown and normalized by the AD‐sham group (c–g). **p < .01,*p < .05

It is reported that rTMS is helpful to alleviate cognitive impairments. However, there are few reports on whether rTMS affects hallmark pathological changes in AD progress such as neuronal Aβ‐containing senile plaque, tau hyperphosphorylation, and APP. Owing to the Aβ‐containing senile plaques cannot be observed until 6 months old in APP/PS1 mouse model, here we used Western blots to detect the Aβ toxicity and APP expression in the hippocampus of mice. The results showed that rTMS significantly reduced the expression of Aβ (Figure 3a,c) and APP (Figure 3a,d). Meanwhile, a similar result was observed in p‐Tau expression (Figure 3b,e–g). These results suggest that rTMS treatment relieved the progression of AD pathology in APP/PS1 mice.

### 2.9 Effect of rTMS on BDNF/SYN/PSD95 in the hippocampus of AD mice

**FIGURE 4:**
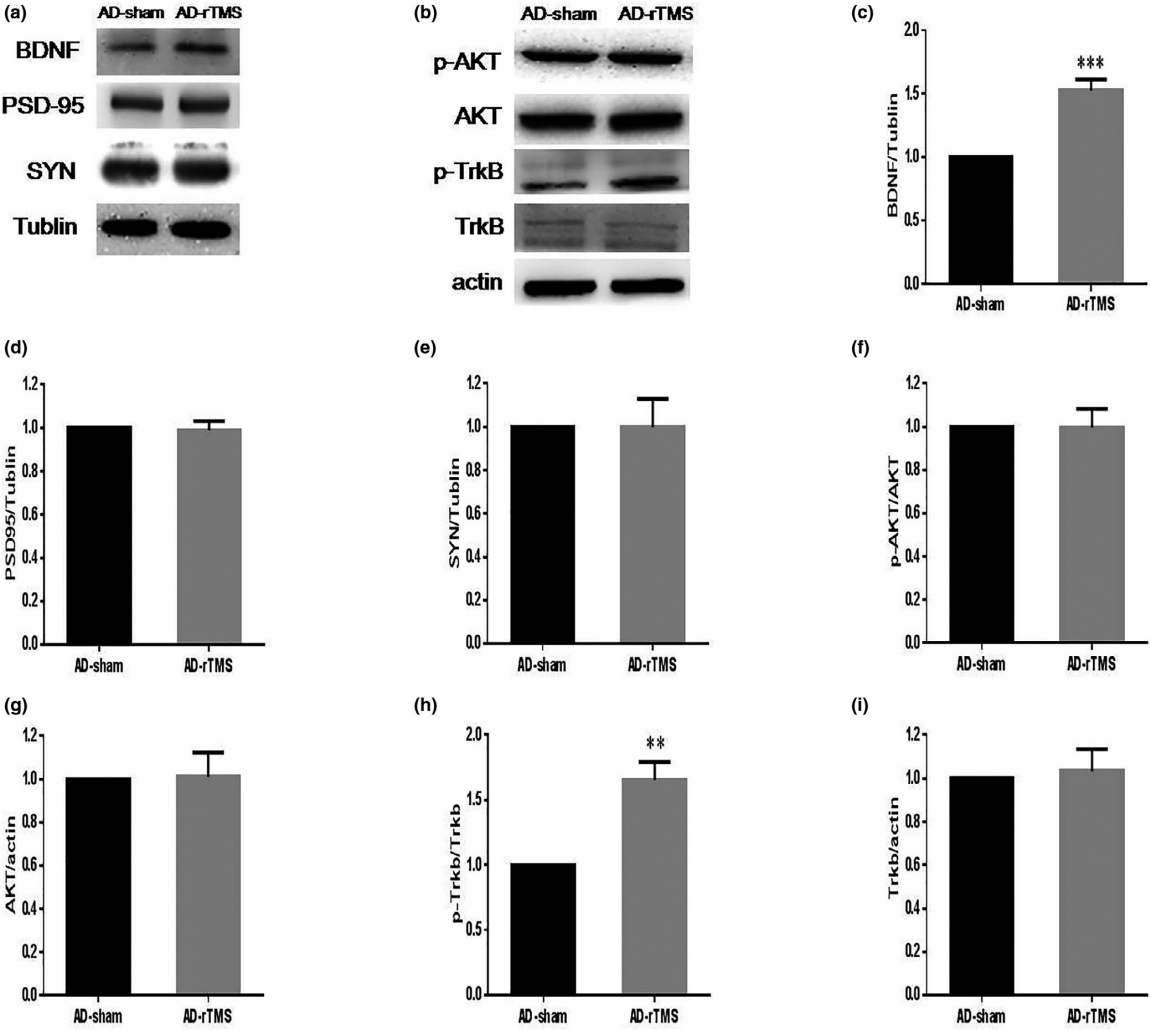
Effects of HF rTMS on BDNF/SYN/PSD95 in the hippocampus. Hippocampal protein expression of BDNF/SYN/PSD95 was determined by Western blots (a). The expression of p‐TrkB and p‐AKT in the downstream pathway of BDNF was also shown by Western blots (b). The quantification graph was shown and normalized by the AD‐sham group (c–i). ***p < .001, **p < .01, *p < .05

Synaptic plasticity‐related proteins (PSD95 and SYN) play an important role in learning and memory function of hippocampus. In addition, BDNF is the most abundant neurotrophic factor in the body, which is the key factor in learning, memory, and cognitive function. To explore the mechanism of rTMS on AD, Western blots were used to detect the changes of these three proteins. The results showed that the expressions of PSD‐95 and SYN remained comparable between the two groups (Figure 4a,d,e), whereas the level of BDNF in the AD‐rTMS group was substantially higher than the AD‐sham group (Figure 4a,c). We therefore investigated the effects on BDNF‐associated signaling downstream pathway: TrkB and AKT. Western blots showed that the level of p‐TrkB was significantly increased in AD‐rTMS mice compared with AD‐sham animals, while the p‐AKT level remained unchanged (Figure 4b,f–i). These results imply that BDNF participated in the treatment of AD mice by rTMS, but there was possible other unknown molecular mechanisms involved.

### 2.10 Improvement of rTMS on the APOE/PP2A signaling pathway and autophagy

**FIGURE 5:**
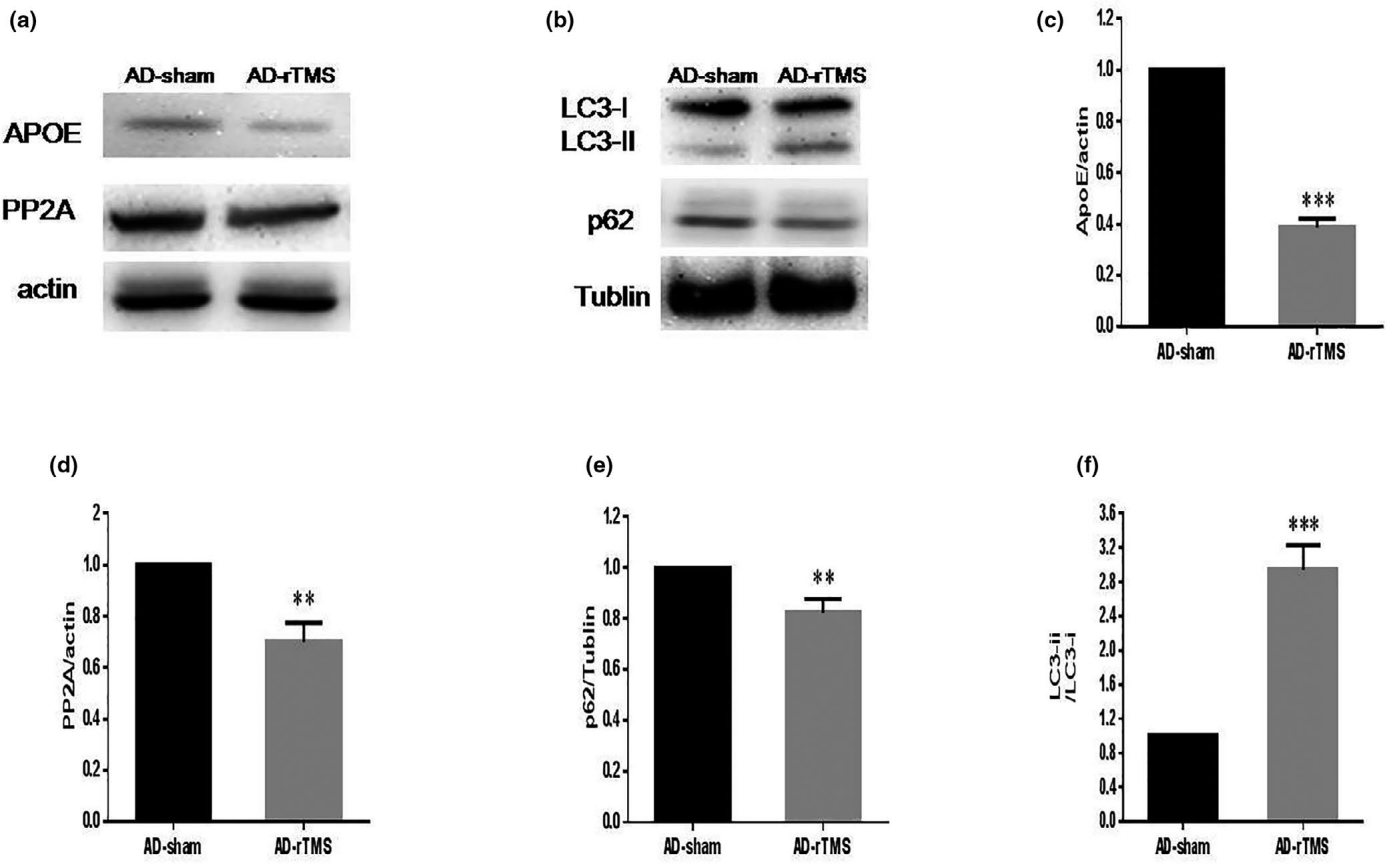
Regulation of rTMS on the APOE/PP2A signaling pathway and autophagy. The expression of ApoE and PP2A in the mouse brain was measured (a). The expression levels of autophagy marker proteins, p62 and lc3ii/lc3i, were also compared by Western blots (b). The quantification graph was shown and normalized by the AD‐sham group (c–f). ***p < .001, **p < .01, *p < .05

ApoE is one of the greatest genetic risk factors for AD. ApoE and its receptor play an important role in the regulation of Aβ imbalance in the early stage of AD. To further study the mechanism underlying rTMS treatment of AD, the expression of ApoE was measured by Western blots. To our interest, the results showed that the expression of ApoE in the brain decreased dramatically after rTMS treatment (Figure 5a,c), and the expression of PP2A was reduced as well (Figure 5a,d).Then, the levels of autophagy marker proteins, p62 and lc3ii/lc3i, were detected. Meanwhile, the level of lc3ii/lc3i in the AD‐rTMS group was significantly increased, accompanied by the decreased expression of p62 (Figure 5b,e,f). These data suggest that rTMS enhanced the hippocampal autophagy level in APP/PS1 mice by down‐regulating ApoE in AD mice.

## 3 DISCUSSION

In this article, we systematically studied the neuroprotective effects of rTMS against AD by APP/PS1 mice. We found that HF rTMS significantly rescues the learning, memory, and cognitive impairment, and reduces the neuropathology associated with AD in APP/PS1 mice. Besides of BDNF‐TrkB signaling, our results show the new insight into the APOE‐PP2A and the autophagy system involved in the neuroprotection of rTMS on AD, which provides more experimental evidence for clinical applications of rTMS in the treatment of AD.

Although rTMS has been used to treat AD in clinic, specific treatment schemes are not unified, and the mechanism is unclear. Thus, the research on rTMS to treat AD needs further investigation (Alcalá‐Lozano et al., 2018; Sabbagh et al., 2019; Turriziani et al., 2019).Recent studies have focused on the cognitive impairment of rTMS in AD model mice or patients, and there are few reports on the characteristic pathological changes and the mechanism of AD lesions (Huang et al., 2017; Ma et al., 2017). This paper focused on the early intervention of rTMS on AD‐related pathological indices, especially of Aβ deposition and phosphorylated Tau, to explore the molecular mechanism by which rTMS enhanced the cognitive function of AD mice.

To date, the removal of abnormal pathogenic proteins (Aβ deposition and phosphorylated Tau protein accumulation) in the brain is still the first option for AD treatment (Blennow, Hampel, & Zetterberg, 2014; Yuan, Yidan, Jian, Xiangjian, & Guofeng, 2020). The lysosomal degradation and clearance of Aβ mediated by ApoE signaling is one of the effective pathways to reduce Aβ accumulation in the brain (Bu, 2009; Langlois et al., 2019). An fMRI study of nondemented elderly found that rTMS intervention led to changes in the brain network connections with different ApoE subtypes of elderly carriers, and improved memory performance slightly as well, in which ε4 subtype was the most obvious effector (Peña‐Gomez et al., 2012). Thus, is the therapeutic effect of rTMS on AD related to ApoE mediated Aβ degradation? Consistent with our results, the expression of ApoE in the brain of APP/PS1 mice was significantly down‐regulated after rTMS intervention. More than that the autophagy level was also increased, and AD‐related pathogenic marker proteins like Aβ, p‐Tau, and their downstream molecules were correspondingly reduced.

In conclusion, HF rTMS intervention for two weeks can restore early AD‐like dysfunctions in the brain of APP/PS1 mice by regulation of APOE and autophagy, which provides a new idea and experimental basis for the early prevention and treatment of AD.

## CONFLICT OF INTEREST

The authors declare that there is no conflict of interest.

## AUTHOR CONTRIBUTION

Lin‐Xiao Wang contributed to the conception of the study. Xia Chen performed the experiments and wrote the manuscript. Guo‐Ying Dong helped perform the data analysis.

### Peer Review

The peer review history for this article is available at https://publons.com/publon/10.1002/brb3.1740.

## Data Availability

The data used to support the findings of this study are available from the corresponding author upon request.
